# Effects of Frequency Drift on the Quantification of Gamma-Aminobutyric Acid Using MEGA-PRESS

**DOI:** 10.1038/srep24564

**Published:** 2016-04-15

**Authors:** Shang-Yueh Tsai, Chun-Hao Fang, Thai-Yu Wu, Yi-Ru Lin

**Affiliations:** 1 Graduate Institute of Applied Physics, National Chengchi University, Taipei, Taiwan; 2 Research Center for Mind, Brain and Learning, National Chengchi University, Taipei, Taiwan; 3 Department of Electronic and Computer Engineering, National Taiwan University of Science and Technology, Taipei, Taiwan

## Abstract

The MEGA-PRESS method is the most common method used to measure γ-aminobutyric acid (GABA) in the brain at 3T. It has been shown that the underestimation of the GABA signal due to B0 drift up to 1.22 Hz/min can be reduced by post-frequency alignment. In this study, we show that the underestimation of GABA can still occur even with post frequency alignment when the B0 drift is up to 3.93 Hz/min. The underestimation can be reduced by applying a frequency shift threshold. A total of 23 subjects were scanned twice to assess the short-term reproducibility, and 14 of them were scanned again after 2–8 weeks to evaluate the long-term reproducibility. A linear regression analysis of the quantified GABA versus the frequency shift showed a negative correlation (P < 0.01). Underestimation of the GABA signal was found. When a frequency shift threshold of 0.125 ppm (15.5 Hz or 1.79 Hz/min) was applied, the linear regression showed no statistically significant difference (P > 0.05). Therefore, a frequency shift threshold at 0.125 ppm (15.5 Hz) can be used to reduce underestimation during GABA quantification. For data with a B0 drift up to 3.93 Hz/min, the coefficients of variance of short-term and long-term reproducibility for the GABA quantification were less than 10% when the frequency threshold was applied.

It is known that γ-aminobutyric acid (GABA) is a primary inhibitory neurotransmitter in the central nervous system. Previous studies have shown that the GABA concentrations in the brain are related to the brain activity measured by MEG and fMRI^1^,^2^,^3^,^4^ and are linked to behavior responses^5^,^6^. GABA has also been found to be related to various neuronal diseases^7^,^8^,^9^. Currently, a spectral editing MRS technique called the MEGA-PRESS (MEscher–GArwood Point RESolved Spectroscopy) sequence is the most common approach used to quantify GABA at 3T^10^,^11^,^12^,^13^. In the context of MEGA-PRESS, one dataset is collected by applying frequency-selective editing pulses at 1.9 ppm to edit coupled spins of GABA at 3 ppm (often referred to as “edit-on”). To provide a different editing scheme, another dataset is collected by applying editing pulses at a symmetrical location to water (often referred to as “edit-off”). These two datasets are collected in an interleaved manner. Subtraction of the “edit-off” from the “edit-on” spectrum removes all peaks unaffected by the editing pulse from the spectrum and retains those affected by the editing pulses. Therefore, the GABA signal at 3.0 ppm and combined glutamate and glutamine (Glx) signals at 3.75 ppm, coupled to Glx at 2.1 ppm, can be observed on the edited spectrum. One limitation of this method is the presence of a co-editing macromolecule (MM) signal at 3.0 ppm due to coupling to the signal at 1.7 ppm by the editing pulse. To address the existence of the contribution from MM, the quantified GABA signal is labeled as GABA+^14^. The observation and quantification of a reliable GABA+ signal relies on post-processing strategies^15^,^16^,^17^,^18^,^19^,^20^ and quantification strategies, which can be performing by integration, fitting^17^,^21^ or commercial software packages such as LCModel^15^,^22^. The performance of quantification strategies has been evaluated in several studies^16^,^23^ with the reproducibility of GABA quantification reported for different brain regions^15^,^17^,^21^,^22^,^24^

Due to the low concentration of GABA, the voxel size of MEGA-PRESS is usually set as large as possible to cover the region of interest, with the number of measurements usually over 256 to ensure that the signal-to-noise ratio (SNR) is in the editing spectrum. In addition, shimming and frequency adjustment need to be carefully performed before data acquisition to ensure that the editing pulses are applied within the presumed spectral range. However, B0 field drift may occur during successive scans, especially after scans involving the heavy use of gradients, such as EPI^19^. This can lead to errors in the quantification of the GABA signal. The sources of errors are attributed to the subtraction artifacts from the misalignment of the edit-on and edit-off spectra and to the changes in the editing efficiency of GABA and MM^19^. A post-processing correction step can be used to minimize the misalignment artifacts. Evens *et al.* showed that pairwise alignment can result in less than 1% error at a B0 drift up to 0.3 ppm/h, which is approximately 0.62 Hz/min^20^. The repeatability of quantifying GABA+/H_2_O can thus be 6% for the occipital lobe. Harris *et al.* further showed that the B0 drift can be more serious for MRS experiments after fMRI scanning^19^. Before fMRI scanning, the B0 drift is less than 0.1 Hz/min, but this increases to −1.22 Hz/min just after fMRI scans are obtained. Even more than 30 min after fMRI scanning, a B0 drift of −0.5 Hz/min was found. In these previous studies, the underestimation of the GABA+ signal due to B0 drift could be reduced by post frequency alignment. However, when the B0 drift is above this level, it is possible that an underestimation of the quantified GABA signal can occur even with post frequency alignment.

In this study, we investigated the GABA quantification with a B0 drift greater than −1.22 Hz/min. The underestimation of GABA still occurred even when subtraction artifacts were minimized by the frequency alignment. To reduce the underestimation, a frequency shift threshold was applied to exclude spectra over the threshold from the analysis. The level of underestimation in the GABA+ quantification was evaluated by a linear regression analysis of the quantified GABA+ versus the frequency shift. An optimized frequency shift threshold was determined based on a regression analysis of nine frequency shift thresholds (0.075 ppm to 0.275 ppm). Finally, the short-term and long-term reproducibility of GABA+ quantification using the optimized frequency shift threshold were determined.

## Results

**Figure 1:**
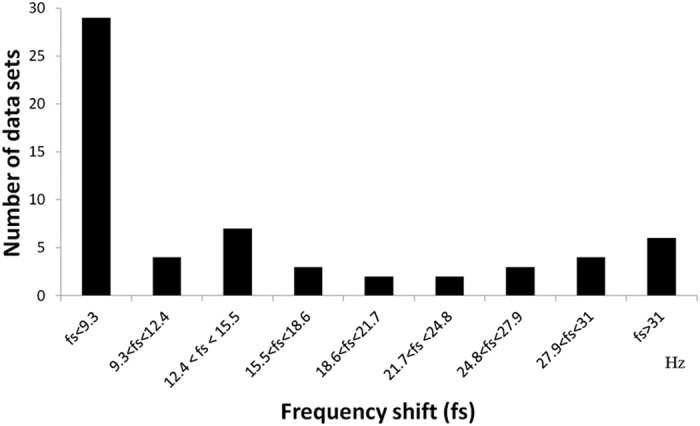
The range of the frequency shifts across the MRS scans for 60 datasets from 23 subjects. The largest frequency shift in this study was 34.1Hz, which is 3.93 Hz/min.

**Figure 2:**
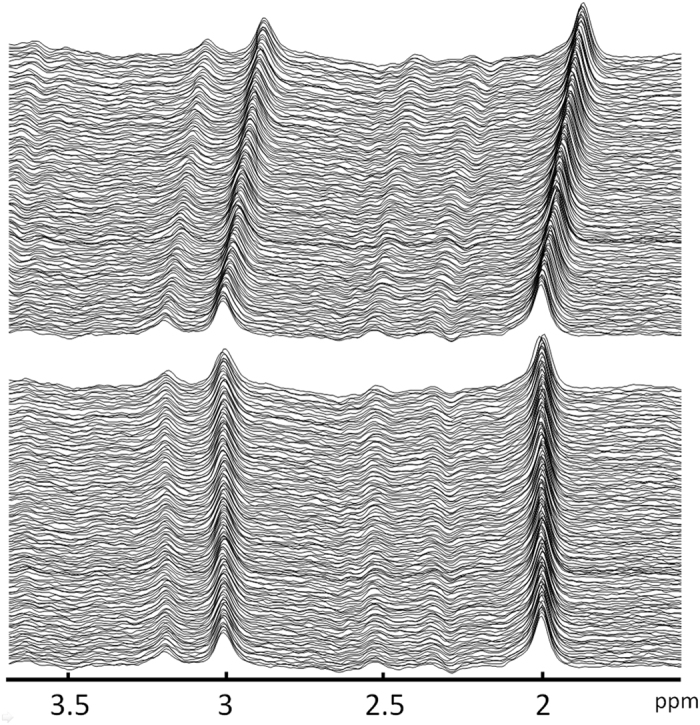
(Top) The 128 edit-off spectra from one subject. (Bottom) Aligned edit-off spectra obtained using post-processing procedures based on the Cr peak.

**Figure 3:**
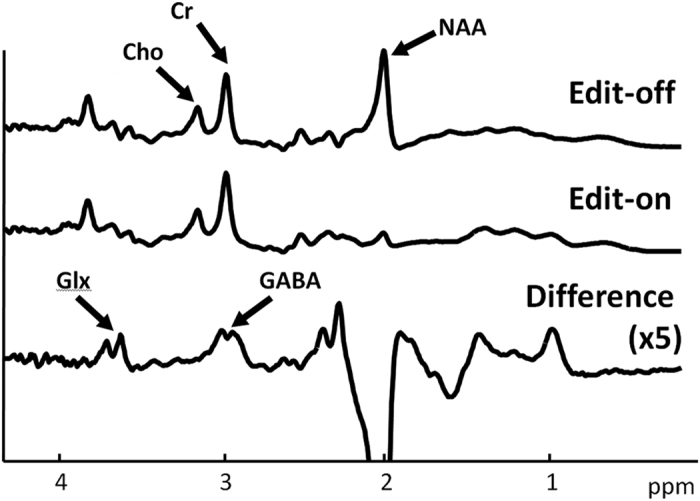
Averaged edit-on, edit-off and edited spectra (the difference between the edit-on and edit-off data) from the same subject shown in Fig. 3. The inverted NAA peak, Glx peaks and GABA peak can be found at 2.02 ppm, 3.75 ppm and 3.0 ppm, respectively, on the edited spectrum.

The Full Width Half Maximums (FWHM) of the water signal for 60 datasets from 23 subjects were 8.55 ± 0.42 Hz. This shows that the field homogeneity can be adjusted to similar conditions. A summary of the frequency shifts across the MRS scans for 60 datasets is shown in Fig. 1. The frequency drift ranged from 0 Hz to 34.1 Hz (3.93 Hz/min). The average B0 drift was 15.9 Hz in scan 1 and was 12.8 Hz in scan 2. The difference was 3 Hz (p > 0.05), as determined using a paired t-test. For the group that underwent scanning with the MRS protocol with minimized B0 drift, the frequency drifts of the six subjects ranged from 0 to 5.86 Hz (3.58 ± 2.56 Hz), which corresponds to 0 Hz/min to 0.68 Hz/min. The edit-off spectra before and after alignment based on the Cr peak from one subject are shown in Fig. 2. The average edit-on, average edit-off and edited spectra are shown in Fig. 3. The typical shapes of the NAA, Cr, and choline spectra can be clearly identified on the edit-off spectrum and the saturated NAA peak can be found on the edit-on spectrum. On the edited spectrum, an inverted NAA peak, Glx peaks and GABA peak can be found at 2.02 ppm, 3.75 ppm and 3.0 ppm, respectively.

**Figure 4:**
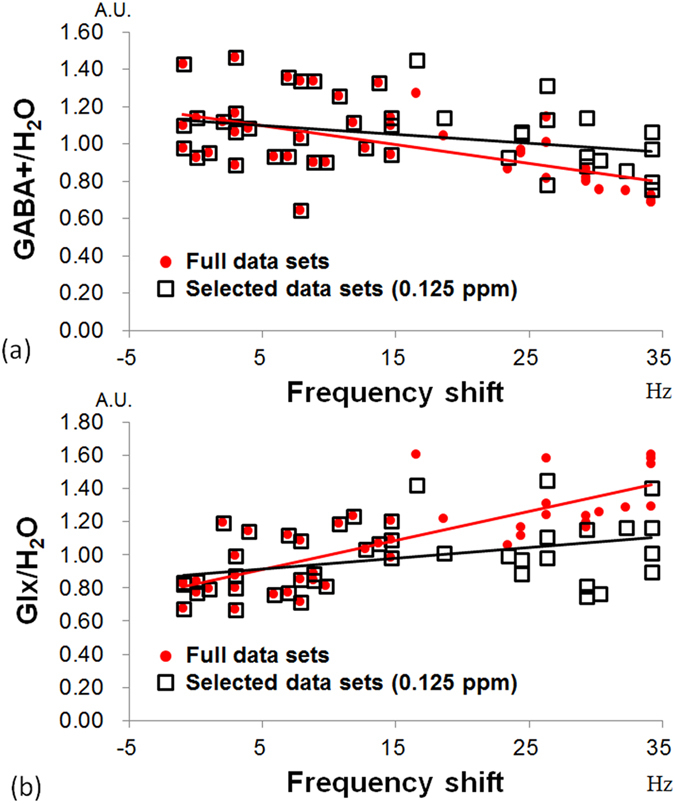
The scatter plot and linear regression of (**a**) GABA+/H_2_O and (**b**) Glx/H_2_O versus the frequency shift. For selected datasets, GABA+/H_2_O and Glx/H_2_O were quantified using the frequency shift threshold at 0.125 ppm (black squares). The slope was −0.0047 for GABA+/H_2_O and 0.0064 for Glx/H_2_O (black line). For full datasets, GABA+/H_2_O and Glx/H_2_O were quantified without a frequency shift threshold (red circles). The slope was −0.0101 for GABA+/H_2_O and 0.0175 for Glx/H_2_O (red line).

**Figure 5:**
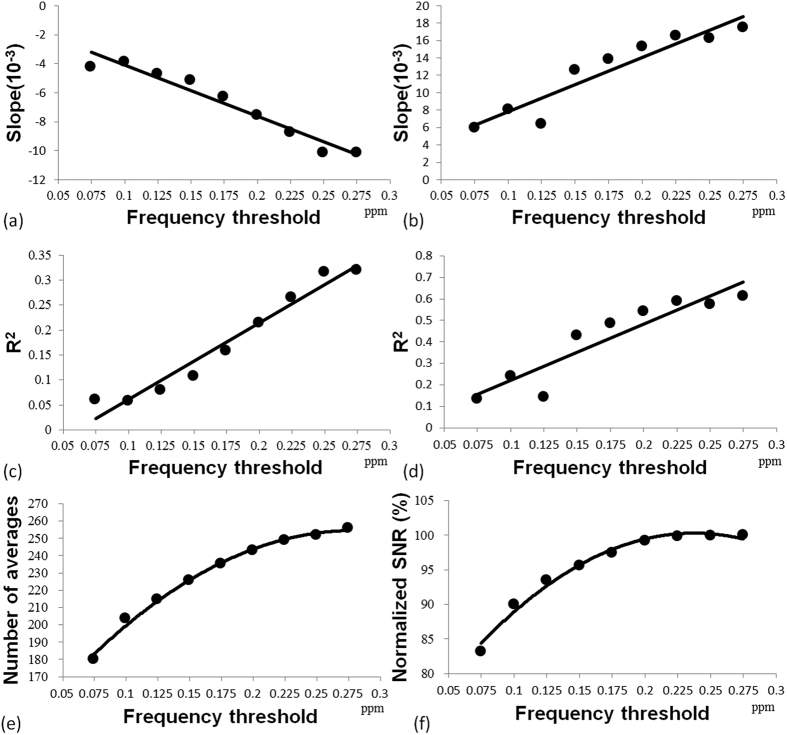
The results of the linear regression analysis (slope and R^2^) using a frequency shift threshold from 0.075 ppm to 0.275 ppm (**a,c**) for GABA+/H_2_O and (**b,d**) for Glx/H_2_O. (**e**) The numbers of averages and (**f**) normalized SNR at the nine frequency thresholds. For GABA+/H_2_O, the regression line showed a statistically significant correlation, with P < 0.01 for the frequency shift threshold at 0.175 ppm to 0.275 and with P < 0.05 for the frequency shift threshold at 0.15 ppm. For Glx/H_2_O, the regression line showed a statistically significant correlation with P < 0.01 for the frequency shift threshold at 0.1 ppm to 0.275 ppm and with P < 0.05 for a frequency shift threshold at 0.075 ppm.

The quantified GABA+/H_2_O from the full datasets without excluding any spectra and from selected datasets with the frequency shift threshold set at 0.125 ppm (15.5 Hz) were plotted versus the frequency shift (Fig. 4a). For the datasets with a frequency shift less than 0.125 ppm (15.5 Hz), the GABA+/H_2_O of the selected datasets and full datasets were identical because no spectra were excluded. For the datasets with a frequency shift over 0.125 ppm (15.5 Hz), the GABA+/H_2_O from selected datasets are higher than those from the full datasets. For the full datasets, the regression line of the plot of GABA+/H_2_O versus the frequency shift showed a negative correlation (P < 0.001). For selected datasets, there was no significant correlation (P = 0.086) (Fig. 4a). On the other hand, the regression line of Glx/H_2_O versus the frequency shift showed a significant positive correction (P < 0.001) for both the full datasets and selected datasets (Fig. 4b). The slope and R^2^ from the linear regression analysis of GABA+/H_2_O and Glx/H_2_O versus the frequency shift for nine frequency shift thresholds (0.075 ppm to 0.275 ppm) are summarized in Fig. 5. The regression lines of the datasets obtained using smaller frequency shift thresholds had smaller R^2^ values and slopes close to 0. The linear regression analysis of GABA+/H_2_O showed no statistically significant correlation (P > 0.05) when a frequency threshold less than 0.125 ppm (15.5 Hz) was used, which implies that the underestimation of the quantified GABA+/H_2_O was not statistically significant at this threshold. The numbers of averages and the SNR also dropped when a smaller frequency shift threshold was applied (Fig. 5e,f). The number of averages was 214.6 ± 62.4 and the normalized SNR was 93.5% at a frequency shift threshold of 0.125 ppm. There were 40 datasets with a frequency shift less than 15.5 Hz (1.79 Hz/min).

The short- and long-term reproducibility of the quantification of GABA+ by integration and fitting are summarized in Table 1. The tissue composition of the VOI in this study only varied by 3%, and the averaged GM/WM/CSF ratio in this study was 0.61 ± 0.026/0.29 ± 0.024/0.10 ± 0.022. Quantification using fitting and integration gave similar concentrations and CVs. For selected data using a frequency shift threshold of 0.125 ppm (15.5 Hz or 1.79 Hz/min), the short-term CVs (n = 23) of GABA+_pvc_ and GABA+/Cr ranged from 6.7% to 7.5%. The long-term CVs (n = 14) of these data ranged from 8.0% to 9.2%. The short- and long-term reproducibility were further evaluated from MRS data with a frequency shift less than 0.125 ppm, which means that all 256 spectra were enrolled without exclusion using the 0.125 ppm frequency shift threshold. In these data, the short-term (n = 13) CVs ranged from 5.5% to 6.8% and the long-term CVs (n = 8) ranged from 7.3% to 8.0%. For the inter-subject variation, the CV of GABA+_pvc_ was approximately 17.8% and the CV of GABA+/Cr was approximately 12.4%. For all 60 datasets from 23 subjects, the SNR was 153.6 ± 57.4.

## Discussion

In this study, the effect of B0 drift during data acquisition on the quantification of GABA was investigated. We showed that the B0 drift can lead to underestimation of GABA+/H_2_O, even with post frequency alignment (Fig. 4a). This is because editing pulses may drift away from GABA C-3 protons at 1.9 ppm during data acquisition, as has been described in previous reports^19^. Our present results showed that the underestimation of GABA+/H_2_O can be lessened using a frequency shift threshold (Fig. 4a). Based on our regression analyses (Fig. 5a,c), the negative correlation between the quantified GABA+/H_2_O and the frequency shift is not statistically significant (P > 0.05) when a frequency shift threshold less than 0.125 ppm (15.5 Hz or 1.79 Hz/min) was used. Therefore, a frequency threshold of 0.125 ppm was chosen in this study to evaluate the short- and long-term reproducibility.

It has been shown that the B0 drift increases after scans with gradient-intensive acquisitions that involve the EPI sequence^19^. In our short-term reproducibility study, B0 drift was smaller in scan 2 than in scan 1. The difference was approximately 3 Hz. It is possible that the smaller B0 drift in scan 2 was due to the extra 10 minutes of rest for the gradient system compared with scan 1. To minimize the B0 drift for MRS scans, running the MRS scans after scans with gradient-intensive acquisitions should be avoided. This can be done by ensuring the proper arrangement of MRS scans in the protocol. Our results showed that inserting a resting session before a MRS scan can minimize the B0 drift during scans. The B0 drifts were in the range of 0 Hz to 5.86 Hz (0 Hz/min to 0.68 Hz/min) in our study. Because EPI is widely used in many applications, such as fMRI or diffusion analyses, there is good chance that EPI scans will have been performed by investigators during the previous time slot. Therefore, it may be necessary to insert approximately 30 minutes of sessions without gradient-intensive acquisitions before MRS scans are obtained. This is not practical in an extensively used system, in which scans are usually performed on a tight schedule.

In previous studies, the reported frequency shifts were less than 0.6 Hz/min^15^,^17^,^21^,^22^ or up to −1.22 Hz/min after serial fMRI scans^19^. Using the post frequency alignment can effectively reduce the underestimation of the GABA signal for the B0 drift at this range^19^,^20^. In the present study, 27 of the 60 datasets had a frequency shift over 1.43 Hz/min. One possible reason why an extended B0 drift range was found in this study is that the experiments evaluated in this study were performed on a MRI system dedicated for fMRI studies, where 90% of the experiments involved EPI scans. Most MRS experiments were conducted after serial fMRI experiments. There was a previous study that reported a similar frequency drift in this range^25^. Our results also imply that when MRS scans cannot be arranged to avoid the effects from EPI scans, underestimation of the GABA signal due to B0 drift over 0.125 ppm (1.79 Hz/min) can occur. Instead of removing all of the MRS data for a subject, a frequency shift threshold can be used to avoid underestimation at the cost of a decrease in the SNR. The findings of the present study are therefore useful for the application of MEGA-PRESS in a clinical system, where MRS scans usually cannot be arranged to minimize the B0 drift.

For the plot of the quantified GABA+/H_2_O and Glx+/H_2_O versus the frequency shift shown in Fig. 4, the negative slope indicates that there were lower quantified GABA+/H_2_O values for datasets with a larger frequency shift. On the contrary, the finding of a positive slope implies that there was a higher quantified Glx/H_2_O value for datasets with a larger frequency shift. In this study, there was a tendency for there to be a positive slope for the B0 drift during MRS acquisition (Fig. 2), which implies that editing pulses were applied at 1.9 ppm at the beginning and drifted toward 2.1 ppm. The underestimation of the GABA+/H_2_O value in the presence of the B0 drift can be attributed to two main factors. The first is the lower editing efficiency of the GABA 1.9 ppm data. The second is the reduced coediting effect of MM at 1.7 ppm^10^,^11^,^14^. Further, a higher Glx/H_2_O in the presence B0 drift can also be expected. Because the Glx signal on the edited spectrum is coedited from the coupling of the Glx group at 2.1 ppm, moving the editing pulse toward 2.1 ppm can increase the effects of editing on the Glx signal. It is worth implementing real-time frequency correction for the MEGA-PRESS sequence to ensure that there is consistent editing efficiency to obtain stable GABA quantification. This is particularly important when editing pulses are applied interleaved at 1.9 ppm and 1.5 ppm to reduce MM contamination^14^.

In this study, the quantified GABA+ values determined using integration and fitting were similar, which is in accordance with a previous report^21^. The short-term CVs were approximately 7.0% for datasets that used a frequency shift threshold of 0.125 ppm. When datasets with a frequency shift less than 0.125 ppm (15.5 Hz or 1.79 Hz/min) were considered, the short-term CVs were lower (approximately 6.2%). The lower CVs may reflect potential variations in the quantification of GABA+ caused by B0 drift, even when a frequency shift threshold is applied. Nevertheless, our results are in good agreement with the 5.5% short-term CVs reported in the occipital region in a previous study^20^. With regard to the long-term reproducibility, 6.5% variation in the long-term reproducibility on a day with a 2.5-hour interval has been reported in the visual region^24^. Another study performed on the left occipital lobe has reported CVs for the long-term reproducibility of 10% to 14.8%^21^. Partial volume correction was not performed in this study, which may be the reason why the CVs were relatively high. In this study, the CVs of the long-term reproducibility were 8.0% to 9.2%, which is in the range of these two previous reports. The differences in the short-term and long-term reproducibility caused by normalized references (water or Cr) were less than 1.2%, which is also in agreement with previous reports^17^,^20^,^21^. A higher inter-subject CV of the GABA+_pvc_ (17.8%) was found in this study compared to a previous study^24^. However, this value is still within the range of previous reports in the left occipital lobe region (13.3% to 17.8%)^21^.

In conclusion, we have investigated the effects of a frequency shift up to 34.1 Hz (3.93 Hz/min) on the quantification of the GABA signal using a MEGA-PRESS sequence. The regression line between the GABA+ signal and frequency shift showed that B0 drift can cause an underestimation of the GABA+ signal even with a post frequency alignment process. A frequency shift threshold at 0.125 ppm (15.5 Hz or 1.79 Hz/ppm) can be used to reduce the underestimation of the GABA+ signal in the occipital lobe, with CVs for the short- and long-term reproducibility of less than 10%.

## Methods

### Participants and data acquisition

A total of 26 healthy volunteers (age: 22.2 ± 1.7 years old, 13 female and 13 male) were enrolled in this study. Before being included in this study, all participants gave their informed consent to undergo a protocol that was approved by the Research Ethics Committee of National Taiwan University. All experiments were performed in accordance with the approved guidelines. Among all subjects, in 3 subjects we could not observe a GABA signal on the edited spectra. Therefore, 23 subjects (age: 22.3 ± 1.7 years old, 11 female, 12 male) were included in the data analysis. To assess the reproducibility, all subjects were scanned twice without leaving the scanner for the short-term reproducibility study, and 14 subjects were rescanned within a variable time interval (44.3 ± 14.9 days) to assess the long-term reproducibility. To minimize the B0 drift during the MRS scan caused by a protocol change, 6 healthy subjects (age: 23.1 ± 1.3 years old, 3 female and 3 male) were enrolled in an additional study. In this subject group, the same MRS scan was performed after at least 1 hour had passed between scanning sessions.

All experiments were performed with a 3T MR system (Skyra, SIEMENS Medical Solutions, Erlangen, Germany) using a 32-channel phased-array head coil. A high-resolution 3D MPRAGE (Magnetization Prepared Rapid Acquisition Gradient Echo) anatomical scan (TR/TE/FA: 2530 ms/3.03 ms/7 degrees; FOV: 256 × 256 × 176; voxel size: 1 × 1 × 1 mm^3^) was initially acquired for localization of the spectroscopic volume of interest (VOI). For MRS scans, a VOI with a size of 30 mm × 25 mm × 25 mm was manually positioned to cover the left and right occipital lobes. To ensure the quality of the spectra, a MRS pre-scan was carried out using a point resolved spectroscopy (PRESS) sequence (TR/TE = 2000/68 ms, sample points = 2048, bandwidth = 2000 Hz, 16 averages). Spectra were analyzed and displayed online to evaluate the linewidth, water suppression and noise level. Once spectra in the pre-scan were considered to be of acceptable quality, GABA measurements were performed directly using the same adjustment parameters for shimming, resonance frequency and water suppression. A MEGA-PRESS sequence was used for GABA measurements^12^,^13^. A total of 260 spectra were acquired using the following parameters: TR/TE = 2000/68 ms, sample points = 2048, bandwidth = 2000 Hz. Edit-on and edit-off spectra were acquired in an interleaved fashion. GABA-editing was achieved with a 20 ms Gaussian pulse applied at 1.9 ppm for edit-on spectra and at 7.5 ppm for edit-off spectra. In addition, a non-water suppression MRS scan was acquired to obtain an unsuppressed water signal for normalization. The total scan time for MRS acquisition was approximately 10 minutes.

### Data processing

For each subject, MRS data were saved separately and the first 4 spectra were excluded. This resulted in 128 edit-on spectra and 128 edit-off spectra. Data processing was performed in MATLAB (The MathWorks, Natick, USA) using in-house scripts. An exponential line broadening filter and phase correction were applied on each time-domain spectrum. To extract information about the resonance frequency shift during scans, the location of the creatine (Cr) peak at 3.01 ppm was identified and fit with a Lorentzien model on each edit-off spectrum. Pairwise alignment was performed based on the location of the Cr peaks for each edit-on and edit-off spectrum. Further phase correction was applied on averaged edit-on and averaged edit-off spectra by aligning the shapes of the residue water peaks. Finally, an edited spectrum was generated by subtraction of the averaged edit-off spectrum from the averaged edit-on spectrum.

The GABA signal at 3.0 ppm was quantified using integration and fitting after a linear baseline correction. To compensate for the existence of a contribution from MM, the signal was labeled as GABA+. For integration, the GABA+ signal at 3.0 ppm and the Glx signal at 3.75 ppm were quantified by integration in a spectral range of ±0.15 ppm. The Cr signal at 3.03 ppm was quantified by integration in a spectral range of ±0.1 ppm on the edit-off spectrum. The fitting methodology was based on the description published by Edden *et al.*^26^. The GABA signal was fitted with two Gaussian line-shape functions with seven variables in a spectral range of 2.79 ~ 3.55 ppm. The Glx signal at 3.75 ppm was also fitted with two Gaussian line-shape functions in a spectral range of 3.5 ~ 4 ppm. On the edit-off spectrum, the Cr signal at 3.03 ppm was fitted by a Lorentzian line-shape function with five variables using a spectral range of 2.93 ~ 3.13 ppm. The water signal was fitted by a Lorentzian line-shape function on the NWS spectra using a spectral range of 3.8 ~ 5.6 ppm. Quantified GABA signals were normalized to water and Cr, and were denoted as GABA+/H_2_O and GABA+/Cr. GABA+/H_2_O was multiplied by 10,000 to make it more convenient for the display. To perform partial volume correction, MPRAGE images were segmented into gray matter (GM), white matter (WM) and cerebral spinal fluid (CSF) images using the segmentation toolbox provided by SPM8 (www.fil.ion.ucl.ac.uk/spm). Separate images of the three tissue types were generated and scaled in percentages. To determine the tissue composition of the VOI, a self-developed program was used to locate the VOI onto MPRAGE images based on the parameters of the location. The percentages of GM, WM and CSF can be determined for each VOI. The GABA+/H_2_O from integration and fitting were thus corrected for partial volume effects using the following equations:


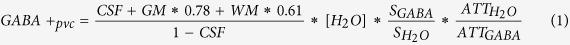



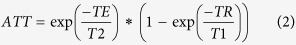


where GABA+_pvc_ is the partial volume-corrected GABA level determined using water as a reference. [H_2_O] is the water concentration in pure water, which is 55.55 M in this case. 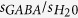 is the GABA+/H_2_O quantified by integration or fitting. ATT was used to account for T1 and T2 relaxation effects using the published relaxation times for GABA and water^27^,^28^,^29^. Fac is a term used to correct the CSF contamination and water density in tissue. Because Cr and GABA have comparable relaxation effects and CSF contamination^22^, GABA+/Cr was not corrected for a partial volume effect.

### Effect of the frequency drift

For each dataset, the frequency shifts across MRS scans were determined based on the location of Cr on the spectrum. To investigate the effects of a frequency drift over 1.22 Hz/min on the quantification of GABA, 9 frequency thresholds ranging from 0.075 ppm to 0.275ppm with 0.025 ppm steps were applied (approximately 9.3 Hz to 34.1 Hz with 3.1 Hz steps). For each subject, all spectra acquired (i.e., measurements) that were over a threshold were removed from the average. Then, 9 MRS datasets were generated by applying the 9 thresholds and the MRS data sets were denoted as “selected datasets.” Notably, no spectrum was excluded for a frequency threshold of 34.1 Hz (0.275 ppm), so the selected dataset with a frequency threshold of 0.275 ppm was referred to as a “full dataset.”

Plots of the quantified GABA+/H_2_O versus the frequency shift were generated for each selected dataset. A linear regression analysis was performed on each plot. The slopes, R^2^ and P-values from the regression analyses were recorded for each frequency shift threshold. The optimized threshold was determined to be when the regression line showed no statistically significant difference (P > 0.05) between the quantified GABA+/H_2_O and frequency shift and the largest SNR retained. For each selected dataset, the numbers of averages (measurements) after excluding spectra and the spectral SNR were calculated for the MRS data from all subjects. The spectral SNR was calculated using the N-Acetyl Aspartate (NAA) peak on averaged edit-off spectra. The NAA signal was fitted using a single Lorentzian line shape. Noise was defined as the standard deviation of the spectral range between 6.7 ppm and 8.5 ppm.

### Reproducibility analysis

Selected datasets with frequency shift thresholds determined from the regression analyses (0.125 ppm/15.5 Hz) were used to investigate the reproducibility. The short-term and long-term reproducibility of the GABA+/H_2_O and GABA+/Cr from integration and fitting were assessed using the coefficient of variation (CV). CVs were calculated as the root mean squares of the standard deviations of the two measurements divided by the means across all subjects. Inter-subject variation was investigated using the CV between subjects.

## Additional Information

**How to cite this article**: Tsai, S.-Y. *et al.* Effects of Frequency Drift on the Quantification of Gamma-Aminobutyric Acid Using MEGA-PRESS. *Sci. Rep.* **6**, 24564; doi: 10.1038/srep24564 (2016).

## Figures and Tables

**Table 1:** The quantified GABA+_pvc_ obtained by integration and fitting using water and Cr as references.

	Short-term reproducibility			Long-term reproducibility			Inter-subject variation		
	n	GABA+_pvc_	GABA+/Cr	n	GABA+_pvc_	GABA+/Cr	N	GABA+_pvc_	GABA+/Cr
Integration^a^	23	0.84 ± 0.06 (6.7)	0.28 ± 0.021 (7.5)	14	0.85 ± 0.08 (9.2)	0.27 ± 0.022 (8.1)	23	0.84 ± 0.15 (17.6)	0.28 ± 0.033 (11.9)
Fitting^a^	23	0.84 ± 0.06 (6.9)	0.28 ± 0.020 (7.3)	14	0.86 ± 0.08 (9.0)	0.28 ± 0.022 (8.0)	23	0.85 ± 0.16 (18.8)	0.28 ± 0.037 (13.3)
Integration^b^	13	0.86 ± 0.05 (6.0)	0.27 ± 0.019 (6.8)	8	0.88 ± 0.07 (8.3)	0.28 ± 0.020 (7.3)	13	0.88 ± 0.15 (16.7)	0.27 ± 0.032 (11.9)
Fitting^b^	13	0.86 ± 0.05 (5.5)	0.28 ± 0.017 (6.2)	8	0.88 ± 0.07 (8.0)	0.28 ± 0.022 (7.7)	13	0.88 ± 0.16 (18.2)	0.27 ± 0.034 (12.6)

The short-term and long-term reproducibility and inter-subject variation are presented as the means ± standard deviation (CV). All values were quantified using a frequency threshold of 0.125 ppm, which is 15.5 Hz, in this study. ^a^The results from all subjects. ^b^The results from subjects with a frequency shift less than 0.125 ppm, which means that all 256 spectra for a MRS dataset were enrolled without exclusion by the frequency shift threshold.

## References

[b1] Muthukumaraswamy S. D., Evans C. J., Edden R. A., Wise R. G. & Singh K. D. Individual variability in the shape and amplitude of the BOLD-HRF correlates with endogenous GABAergic inhibition. Human brain mapping 33, 455–465, doi: 10.1002/hbm.21223 (2012). 21416560 PMC3374935

[b2] Boy F. *et al.* Individual differences in subconscious motor control predicted by GABA concentration in SMA. Current biology: CB 20, 1779–1785, doi: 10.1016/j.cub.2010.09.003 (2010). 20888227 PMC3128986

[b3] Muthukumaraswamy S. D., Edden R. A., Jones D. K., Swettenham J. B. & Singh K. D. Resting GABA concentration predicts peak gamma frequency and fMRI amplitude in response to visual stimulation in humans. Proc Natl Acad Sci USA 106, 8356–8361, doi: 10.1073/pnas.0900728106 (2009). 19416820 PMC2688873

[b4] Edden R. A., Muthukumaraswamy S. D., Freeman T. C. & Singh K. D. Orientation discrimination performance is predicted by GABA concentration and gamma oscillation frequency in human primary visual cortex. The Journal of neuroscience: the official journal of the Society for Neuroscience 29, 15721–15726, doi: 10.1523/JNEUROSCI.4426-09.2009 (2009). 20016087 PMC6666191

[b5] Jocham G., Hunt L. T., Near J. & Behrens T. E. A mechanism for value-guided choice based on the excitation-inhibition balance in prefrontal cortex. Nature neuroscience 15, 960–961, doi: 10.1038/nn.3140 (2012). 22706268 PMC4050076

[b6] Long Z. *et al.* Thalamic GABA predicts fine motor performance in manganese-exposed smelter workers. PLos One 9, e88220, doi: 10.1371/journal.pone.0088220 (2014). 24505436 PMC3913772

[b7] Gaetz W. *et al.* GABA estimation in the brains of children on the autism spectrum: measurement precision and regional cortical variation. NeuroImage 86, 1–9, doi: 10.1016/j.neuroimage.2013.05.068 (2014). 23707581 PMC3883951

[b8] Shin Y. W. *et al.* Increased resting-state functional connectivity between the anterior cingulate cortex and the precuneus in panic disorder: resting-state connectivity in panic disorder. Journal of affective disorders 150, 1091–1095, doi: 10.1016/j.jad.2013.04.026 (2013). 23688914 PMC3759545

[b9] Emir U. E., Tuite P. J. & Oz G. Elevated pontine and putamenal GABA levels in mild-moderate Parkinson disease detected by 7 tesla proton MRS. PLos One 7, e30918, doi: 10.1371/journal.pone.0030918 (2012). 22295119 PMC3266292

[b10] Terpstra M., Marjanska M., Henry P. G., Tkac I. & Gruetter R. Detection of an antioxidant profile in the human brain *in vivo* via double editing with MEGA-PRESS. Magn Reson Med 56, 1192–1199, doi: 10.1002/mrm.21086 (2006). 17089366

[b11] Terpstra M., Ugurbil K. & Gruetter R. Direct *in vivo* measurement of human cerebral GABA concentration using MEGA-editing at 7 Tesla. Magn Reson Med 47, 1009–1012, doi: 10.1002/mrm.10146 (2002). 11979581

[b12] Mescher M., Merkle H., Kirsch J., Garwood M. & Gruetter R. Simultaneous *in vivo* spectral editing and water suppression. NMR Biomed 11, 266–272 (1998). 9802468 10.1002/(sici)1099-1492(199810)11:6<266::aid-nbm530>3.0.co;2-j

[b13] Mullins P. G. *et al.* Current practice in the use of MEGA-PRESS spectroscopy for the detection of GABA. NeuroImage 86, 43–52, doi: 10.1016/j.neuroimage.2012.12.004 (2014). 23246994 PMC3825742

[b14] Edden R. A., Puts N. A. & Barker P. B. Macromolecule-suppressed GABA-edited magnetic resonance spectroscopy at 3T. Magn Reson Med 68, 657–661, doi: 10.1002/mrm.24391 (2012). 22777748 PMC3459680

[b15] O’Gorman R. L., Michels L., Edden R. A., Murdoch J. B. & Martin E. *In vivo* detection of GABA and glutamate with MEGA-PRESS: reproducibility and gender effects. J Magn Reson Imaging 33, 1262–1267, doi: 10.1002/jmri.22520 (2011). 21509888 PMC3154619

[b16] Henry M. E., Lauriat T. L., Shanahan M., Renshaw P. F. & Jensen J. E. Accuracy and stability of measuring GABA, glutamate, and glutamine by proton magnetic resonance spectroscopy: a phantom study at 4 Tesla. J Magn Reson 208, 210–218, doi: 10.1016/j.jmr.2010.11.003 (2011). 21130670 PMC4641575

[b17] Geramita M. *et al.* Reproducibility of prefrontal gamma-aminobutyric acid measurements with J-edited spectroscopy. NMR Biomed 24, 1089–1098, doi: 10.1002/nbm.1662 (2011). 21290458

[b18] Waddell K. W., Avison M. J., Joers J. M. & Gore J. C. A practical guide to robust detection of GABA in human brain by J-difference spectroscopy at 3 T using a standard volume coil. Magn Reson Imaging 25, 1032–1038, doi: 10.1016/j.mri.2006.11.026 (2007). 17707165 PMC2131736

[b19] Harris A. D. *et al.* Impact of frequency drift on gamma-aminobutyric acid-edited MR spectroscopy. Magn Reson Med 72, 941–948, doi: 10.1002/mrm.25009 (2014). 24407931 PMC4017007

[b20] Evans C. J. *et al.* Subtraction artifacts and frequency (mis-)alignment in J-difference GABA editing. J Magn Reson Imaging 38, 970–975, doi: 10.1002/jmri.23923 (2013). 23188759

[b21] Bogner W. *et al.* *In vivo* quantification of intracerebral GABA by single-voxel (1)H-MRS-How reproducible are the results? Eur J Radiol 73, 526–531, doi: 10.1016/j.ejrad.2009.01.014 (2010). 19201120

[b22] Harada M., Kubo H., Nose A., Nishitani H. & Matsuda T. Measurement of variation in the human cerebral GABA level by *in vivo* MEGA-editing proton MR spectroscopy using a clinical 3 T instrument and its dependence on brain region and the female menstrual cycle. Human brain mapping 32, 828–833, doi: 10.1002/hbm.21086 (2011). 20645307 PMC6870297

[b23] Kaiser L. G., Young K., Meyerhoff D. J., Mueller S. G. & Matson G. B. A detailed analysis of localized J-difference GABA editing: theoretical and experimental study at 4 T. NMR Biomed 21, 22–32, doi: 10.1002/nbm.1150 (2008). 17377933

[b24] Evans C. J., McGonigle D. J. & Edden R. A. Diurnal stability of gamma-aminobutyric acid concentration in visual and sensorimotor cortex. J Magn Reson Imaging 31, 204–209, doi: 10.1002/jmri.21996 (2010). 20027589

[b25] Wu M. L. *et al.* Frequency stabilization using infinite impulse response filtering for SSFP fMRI at 3T. Magn Reson Med 57, 369–379, doi: 10.1002/mrm.21138 (2007). 17260379

[b26] Edden R. A., Puts N. A., Harris A. D., Barker P. B. & Evans C. J. Gannet: A batch-processing tool for the quantitative analysis of gamma-aminobutyric acid–edited MR spectroscopy spectra. J Magn Reson Imaging. 40, 1445–1452, doi: 10.1002/jmri.24478 (2014). 25548816 PMC4280680

[b27] Gasparovic C. *et al.* Use of tissue water as a concentration reference for proton spectroscopic imaging. Magn Reson Med 55, 1219–1226 (2006). 16688703 10.1002/mrm.20901

[b28] Puts N. A., Barker P. B. & Edden R. A. Measuring the longitudinal relaxation time of GABA *in vivo* at 3 Tesla. J Magn Reson Imaging 37, 999–1003, doi: 10.1002/jmri.23817 (2013). 23001644 PMC3531569

[b29] Edden R. A., Intrapiromkul J., Zhu H., Cheng Y. & Barker P. B. Measuring T2 *in vivo* with J-difference editing: application to GABA at 3 Tesla. J Magn Reson Imaging 35, 229–234, doi: 10.1002/jmri.22865 (2012). 22045601 PMC3377980

